# Opioid equipotency conversions for hospitalized infants: a systematic review

**DOI:** 10.1038/s41372-024-02121-z

**Published:** 2024-09-20

**Authors:** Madeleine C. Ing, Olivia A. Keane, Ashwini Lakshmanan, Eugene Kim, Henry C. Lee, Lorraine I. Kelley-Quon

**Affiliations:** 1 Division of Pediatric Surgery, Children’s Hospital Los Angeles, Los Angeles, California USA; 2 Department of Surgery, Keck School of Medicine, University of Southern California, Los Angeles, CA USA; 3 Department of Health Systems Science, Bernard J. Tyson Kaiser Permanente School of Medicine, Pasadena, CA USA; 4 Division of Pain Medicine, Children’s Hospital Los Angeles, Los Angeles, CA USA; 5 Department of Anesthesiology and Critical Care Medicine, Children’s Hospital Los Angeles, Los Angeles, CA USA; 6 Division of Neonatology, University of California San Diego, La Jolla, CA USA; 7 Department of Population and Public Health Sciences, Keck School of Medicine, University of Southern California, Los Angeles, CA USA

**Keywords:** Outcomes research, Paediatrics

## Abstract

Hospitalized infants commonly receive opioids to reduce pain and minimize distress during invasive procedures. However, infant neurodevelopment is significantly impacted by cumulative and prolonged opioid exposures. While opioid conversion has been studied extensively in adults, no standardized equipotency opioid conversions exist for hospitalized infants and opioid stewardship efforts are inconsistent. We performed a systematic review to identify opioid dosing conversions commonly used in hospitalized infants <1 year of age, finding fourteen articles which documented or cited a calculation of cumulative opioid exposure. Morphine milligram equivalents (MME) conversion factors varied widely, with nine studies citing conversion equivalent equations commonly used in adults. Efforts to expand safe opioid stewardship to hospitalized infants will require evidence-based consensus for opioid equipotency dose conversions which acknowledge the unique physiology of infants.

## Introduction

Opioid medications are a mainstay treatment for hospitalized infants. Pain and stress can cause acute physiologic response and morbidity [1] including abnormal brain development [2, 3], neurodevelopment [2, 4], somatosensory [2, 5, 6], and stress responses [2]. However, increased opioid exposure in infants has also been associated with worse neurodevelopmental outcomes [7–9] as well as lower body weight [8], and more social problems [8]. Opioid conversion has been studied extensively in adults and government entities like the Centers for Disease Control and Prevention (CDC) have published conversion tables for opioids [10]. However, there are no consensus-based guidelines to inform opioid dosing in the pediatric population, including the neonatal nor infant populations. Previous reviews have shown wide variation in conversion factors used within the pediatric population [11, 12]. Some pediatric studies have suggested use of different conversion factors for specific opioids [13, 14] though further validation and more pharmacokinetic studies are needed [15]. Importantly, however, these studies excluded the infant and neonatal populations. Furthermore, exposure to opioids within the pediatric infant population varies widely across pediatric institutions [16–20].

Neonates and infants have distinct physiologies that contribute to their responses to analgesic medications. Infants have immature hepatic enzymatic functioning leading to decreased clearance of many drugs metabolized by the liver [21, 22]. Additionally, decreased glomerular filtration in the first 8 to 12 months of life leads to impaired clearance of renally excreted drugs [21, 22]. Neonates have larger body water composition relative to muscle and fat compartments, thus increasing the volume of distribution for water-soluble drugs [21, 22]. Neonates also have fewer plasma binding proteins, such that drugs with high protein binding will have an increased fraction of free drug circulating in neonates [21, 22]. These effects may be even more pronounced in preterm neonates [22]. As it relates to opioids, studies have shown that both morphine [23–25] and fentanyl [26] have increased elimination half-lives in neonates.

Though morphine and fentanyl are the most commonly used opioids for hospitalized infants [27], a wide variety of opioids, including methadone, hydromorphone, and oxycodone, are used for analgesia in this population. In addition, each opioid has a unique pharmacodynamic and pharmacokinetic profile which may impact its short- and long-term effects in infants.

As opioid stewardship becomes increasingly crucial to the delivery of quality healthcare, clinicians caring for hospitalized infants must think carefully about how to integrate its principles into routine practice. Though standard opioid conversion factors have been validated for adult populations [10], it is likely that these ratios would not hold in neonates and infants with distinct physiologies. Nonetheless, several pediatric institutions have embedded automatic tools into their electronic health records which calculate cumulative opioid exposure based on adult conversion factors. Few opioid conversion studies have been performed in pediatric populations to date [28], and these have so far been performed with small sample sizes.

To ensure consistency across studies and in acknowledgment of the unique physiology of infants, calculators dedicated to pediatric and infant populations need to be identified and optimized. The goal of this review was to identify opioid dosing conversions commonly used in studies of infant populations.

## Methods

### Search methods

The search strategy was designed to capture literature in which Morphine Milligram Equivalents (MME) were used to quantify opioid administration in infants. Search terms were developed by authors in conjunction with a medical research librarian. PubMed and Embase databases were systematically searched for articles published between January 1, 2012 and August 1, 2023 using the search terms: (morphine equivalent) AND (neonat* OR NICU OR newborn OR newborns OR infan* OR baby OR babies). We chose to include articles beginning in 2012 to capture the most recent decade of literature. The Preferred Reporting Items for Systematic reviews and Meta-Analyses (PRISMA) guidelines were followed [29]. This study was registered with the International Prospective Register of Systematic Reviews (PROSPERO) prior to search initiation (ID CRD42023482449).

### Eligibility criteria

Research studies from all specializations relating to pediatrics and surgery were included to understand the full scope of practice. The population of interest was infants less than one year of age receiving opioids in the inpatient setting. Studies were eligible if they studied exclusively patients less than one year of age and documented or cited a conversion factor between Morphine Milligram Equivalents (MME). Studies were excluded if they referenced MME but did not [1] define the conversion factor used to quantify MME or [2] cite sources from which they obtained a conversion factor used to quantify MME. Manuscripts published in a language other than English were excluded. Clinical trials, observational studies (case-control and cohort), case series, case studies, quality improvement studies, and review studies were all included. Letters to the editors, editorials, commentaries were excluded.

### Selection process

Two independent reviewers (MI, OAK) screened all titles and abstracts identified by our search strategy. Abstracts meeting inclusion criteria were chosen and full manuscripts were reviewed for exclusion criteria. All studies ultimately included were approved by both independent reviewers. There were no disagreements on final study inclusion criteria.

### Data extraction

Conversion factors, equianalgesic ratios, or equianalgesic doses used to calculate morphine milligram equivalents (MME) were extracted by full manuscript review. In articles which cited other literature documenting a conversion factor for MME, the full manuscript of the cited literature was reviewed to extract a conversion factor. Manuscripts were also reviewed to extract the source of the conversion factors chosen by authors–specifically, if the conversion factors being used in each study were generated based on adult physiology, pediatric physiology, or neonatal physiology. For the purpose of this study, we will refer to these conversion factor source populations as reference populations.

One reviewer extracted data using a standardized data collection template. The second reviewer checked the extracted data. Disagreements were re-evaluated in partnership.

### Synthesis methods

A primary aim of this study was to compare the equianalgesic doses reported to convert between opioid types. With data extracted from manuscripts or cited sources, reviewers calculated doses (in milligrams (mg)) of intravenous (IV) fentanyl, IV hydromorphone, and oral (PO) oxycodone equivalent to 1 mg of IV morphine (Table 1). Reviewers additionally used the extracted data to calculate doses of PO methadone equivalent to 1 mg of PO morphine (Table 2). Data was summarized in Table 3.

**Table 1:** Short-acting opioid doses equivalent to 1 IV morphine milligram equivalent (MME).

Authors	Equivalents used in methodology	EAD documented in text	EAD sources cited	Sources	Reference population of EAD	IV Fentanyl EAD (mg)	IV Hydromorphone EAD (mg)	PO Oxycodone EAD (mg)
Czarnecki et al. [33]	IV Morphine equivalents	Yes	Yes	Authors chose own “conservative” conversion factor based on review of neonatal, pediatric, and adult literature.	Neonatal	0.05		
Lewis et al. [34]	IV Morphine equivalents	Yes	Yes^2^	Saarenmaa et al. [49], Simons et al. [55]	Neonatal	0.05	0.2	
Rana et al. [40]	IV Morphine equivalents	Yes	No^3^	–	Pediatric	0.01		
Martin et al. [35]	IV Morphine equivalents	Yes	Yes	Pereira et al. [56]	Adult	0.01	0.25	
Romnek et al. [37]	PO Morphine equivalents	Yes	Yes	Gammaitoni et al. [47], Guinard et al. 1995 [57]	Adult	0.01*	0.15*	2*
Stetson et al. [42]	IV Morphine equivalents	No	Yes	Brennan et al. [58], ClinCalc.com [59]	Adult	0.01		
Resnick et al. [44]	PO Morphine equivalents	No	Yes	Arnold et al. [60] Gammaitoni et al. [47]	Adult	0.01*		2-3*
Achuff et al. [43]	PO Morphine equivalents	Yes	Yes	Pereira et al. [56], WA State Agency of Medical Directors 2015 [61], Chung et al. [62] Nielsen et al.[63]	Adult	0.03*	0.15*	2*
Gil et al. [38]	PO Morphine equivalents	No	Yes	Patanwala 2007 [46]	Adult	0.01-0.02*		
Vavolizza et al. [32]	IV Morphine equivalents	Yes	No	–	–	0.01		3
Bae et al. [39]	IV Morphine equivalents	Yes	No	–	–	0.01		2

*Reviewers converted the reported conversion factors or equianalgesic doses (EAD)^1^ to 1 IV MME prior to inclusion in the table.

1 EAD Equianalgesic dose(s).

2 The sources listed in this table only referenced fentanyl conversion. No citations were provided for hydromorphone conversion nor methadone conversion (displayed in Table 2).

3 Though Rana et al. cited a source for methadone conversion referenced in Table 2, no citation was provided for the fentanyl conversion documented in text and displayed in this table.

**Table 2:** Methadone doses equivalent to 1 PO morphine milligram equivalent (MME).

Authors	Equivalents used in methodology	EAD documented in text	EAD sources cited	Sources	Reference population of EAD	PO Methadone EAD (mg)
Lewis et al. [24]	IV Morphine equivalents	Yes	No^2^		Neonatal	0.1*
Rana et al. [25]	IV Morphine equivalents	Yes	Yes	Jeffries et al. [28]	Pediatric	1 mg PO methadone = 1 mg IV morphine^3^
Achilles et al. [26]	PO Methadone equivalents	Yes	Yes	Bruera et al. [64]	Adult	0.2857
Taleghani et al. [28]	PO Morphine equivalents	No	Yes	CDC [10]	Adult	0.25
Achuff et al. [32]	PO Morphine equivalents	Yes	Yes	Pereira et al. [56], WA State Agency of Medical Directors 2015 [61]	Adult	0.25
Sutter et al. [34]	PO Morphine equivalents	Yes	Yes	CDC [10]	Adult	0.25

*Reviewers converted the reported conversion factors or equianalgesic doses (EAD)^1^ to 1 PO MME prior to inclusion in the table.

1 EAD Equianalgesic dose(s).

2 Though Lewis et al. cited a source for fentanyl conversion as displayed in Table 2, no citation was provided for methadone conversion.

3 Rana et al. directly converted PO methadone to IV morphine and did not provide any conversion to PO morphine in text. Therefore, reviewers were unable calculate equivalent dose to 1 PO MME and this author’s methadone EAD was excluded from Fig. 2.

**Table 3:** Summary of adult and infant conversion factors cited in the literature.

Drug/Agent	Adult Conversion Equivalents (mg)	Range in Infants from the Literature (mg)
IV Fentanyl	0.01–0.03	0.05
IV Hydromorphone	0.15–0.25	0.2
PO Oxycodone	2–3	–
*PO Methadone	*0.25–0.29	*0.1

Conversion factors listed are equivalent to 1 IV MME except for *PO methadone conversion factors listed are equivalent to 1 PO MME.

## Results

### Study selection

**Fig. 1:**
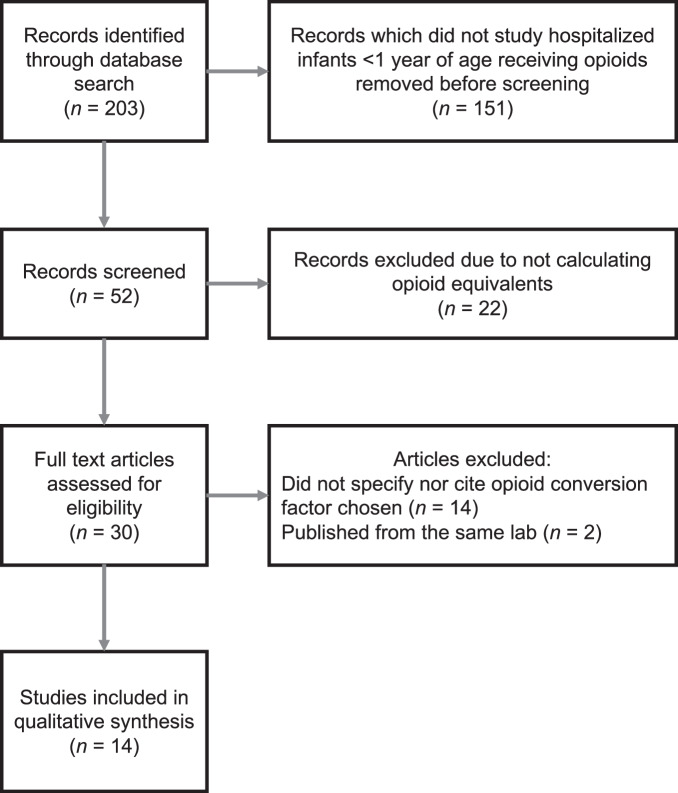
PRISMA flow diagram. Study flow of literature search and exclusions.

The search strategy identified a total of 203 studies (Fig. 1). Of these, 52 focused on infants <1 year of age, and 30 of those studies used opioid equivalents. Fourteen studies were eliminated because they did not document nor cite the equianalgesic doses or conversion factors used. Of the remaining 16 studies, three were published from the same two primary authors (Grabski & Vavolizza [30–32]), therefore two were eliminated to avoid bias of the results [30, 31]. Ultimately, 14 studies were included (Table 4).

**Table 4:** Summary of studies of infants <1 year old in which equianalgesic opioid doses were documented or cited.

Authors	Year	Study type	Patients with ICU level care included	Study limited to patients with exposure to opioids in utero	Sample size	Comparative groups	Risk of bias judgment^1^ (Assessment tool used)
Czarnecki et al. [33]	2014	Single institution retrospective cohort study.	Yes	No	*n* = 33	Parent/nurse-controlled analgesia (*n* = 20) vs continuous opioid infusion (*n* = 13) for postoperative infants in the NICU.	Moderate^2^ (ROBINS-I)
Lewis et al. [34]	2015	Single institution retrospective cross-sectional cohort study.	Yes	No	*n* = 63	Comparison of average cumulative opioid exposure in high-risk infants admitted between 2003-2004 (*n* = 21), between 2007-2008 (*n* = 14), and between 2010-2011 (*n* = 28).	Low risk of bias^3^ (ROBINS-E)
Rana et al. [40]	2017	Single institution QI^4^ study.	Yes	No	*n* = 211	Implementation of pain and medication dosing guidelines for patients undergoing procedures and mechanical ventilation in the NICU. Pre-intervention (*n* = 129) vs post-intervention (*n* = 82).	Moderate^2^ (ROBINS-I)
Achilles et al. [41]	2019	Single institution QI^4^ study.	No	Yes	*n* = 181	Implementation of an Eat, Sleep, Console Assessment Tool in neonates exposed to opioids in utero. Pre-intervention (*n* = 81) vs post-intervention (*n* = 100).	Moderate^2^ (ROBINS-I)
Martin et al. [35]	2019	Single institution retrospective cohort study.	Yes	No	*n* = 82	Epidural analgesia (*n* = 47) vs non-epidural analgesia (*n* = 35) for patients undergoing major abdominal surgery.	Moderate^2^ (ROBINS-I)
Taleghani et al. [36]	2019	Multicenter retrospective cohort study.	Yes – however, comorbidities excluded.	Yes	*n* = 156	Buprenorphine (*n* = 48) vs methadone (*n* = 108) treatment for neonates exposed to long-acting opioids in utero.	Moderate^2^ (ROBINS-I)
Romnek et al. [37]	2020	Single institution retrospective cohort study.	Yes	No	*n* = 28	Minimal invasive surgical (*n* = 8) vs open (*n* = 20) repair of congenital diaphragmatic hernia.	Moderate^2^ (ROBINS-I)
Stetson et al. [42]	2020	Single institution QI^4^ study.	Yes	No	*n* = 222	Implementation of educational sessions, guidelines, and operations coordination to decrease opioid exposure in the NICU. Pre-intervention (*n* = 72), during project planning (*n* = 39), post-intervention (*n* = 111).	Moderate^2^ (ROBINS-I)
Resnick et al. [44]	2021	Single institution retrospective observational study.	Yes	No	*n* = 25	Frequency of postoperative respiratory events after mandibular distraction removal in infants who underwent mandibular distractor osteogenesis.	Low risk of bias^3^ (ROBINS-E)
Achuff et al. [43]	2022	Single institution QI^4^ study.	Yes	No	*n* = 54	Implementation of a standardized opioid weaning protocol using morphine in a pediatric cardiac ICU (*n* = 27). Previous standard of care was non-protocolized methadone wean (*n* = 27).	Moderate^2^ (ROBINS-I)
Gil et al. [38]	2022	Single institution retrospective cohort study.	Yes	No	*n* = 149	Examination of risk factors leading to of patients prolonged ( > 24 hours) (*n* = 102) vs non-prolonged (*n* = 47) extubation following coarctation of aorta repair.	Low risk of bias^3^ (ROBINS-E)
Sutter et al. [45]	2022	Single institution randomized controlled pilot study.	Yes, but only included if NICU stay <24 hours. Comorbidities excluded.	Yes	*n* = 28	Methadone (*n* = 13) vs morphine (*n* = 15) for treatment of Neonatal Opioid Withdrawal Syndrome (NOWS) in neonates exposed to opioids in utero.	Low risk of bias^5^ (RoB 2)
Vavolizza et al. [32]	2022	Single institution QI^4^ study.	No	No	*n* = 131	Implementation of standing IV acetaminophen as baseline analgesia for postoperative infants. Pre-intervention (*n* = 56), during project roll-out (*n* = 17), post-intervention (*n* = 58).	Moderate^2^ (ROBINS-I)
Bae et al. [39]	2023	Single institution retrospective cohort study.	Yes	No	*n* = 91	Epidural analgesia (*n* = 63) vs non-epidural analgesia (*n* = 28) for patients with biliary atresia undergoing Kasai portoenterostomy.	Moderate^2^ (ROBINS-I)

1 Risk of bias was assessed via tools below as recommended by Cochrane guidelines for systematic reviews [65].

a Revised Cochrane risk-of-bias tool for randomized trials (RoB 2) [66].

b Risk of Bias in Non-randomized Studies of Interventions (ROBINS-I) [67].

c Risk of Bias in Non-randomized Studies of Exposures (ROBINS-E) [68].

2 From ROBINS-I tool: Moderate risk of bias is interpreted as the study appears to provide sound evidence for a non-randomized study but cannot be considered comparable to a well-performed randomized trial.

3 From ROBINS-E tool: Low risk of bias except for concerns about uncontrolled confounding due to the nature of observational study.

4 QI Quality improvement.

5 From RoB 2 tool: The study is judged to be at low risk of bias for all domains for this result.

Of the 14 included studies, there were seven retrospective cohort studies [33–39], five quality improvement (QI) studies [32, 40–43], one retrospective observational study [44], and one randomized controlled pilot study [45]. Twelve studies included patients requiring ICU-level care [33–40, 42–45]. Three studies exclusively followed patients exposed to opioids in utero who required medication treatment for opiate withdrawal [36, 41, 45].

### Documentation and citation of conversion factors

Ten studies documented the conversion factors, equianalgesic ratios, or equianalgesic doses used to calculate MMEs within the text of their manuscripts [32–35, 37, 39–41, 43, 45], leaving four studies which only cited outside literature when explaining their MME calculations [36, 38, 42, 44]. Twelve studies cited at least one of their equianalgesic doses [33–38, 40–45] with two studies not citing any sources for the conversion factors reported in text [32, 39].

### Source population of conversion factors

Of the twelve studies which cited the sources of their conversion factors, nine derived their equianalgesic doses from adult reference populations [35–38, 41–45]. Czarnecki et al. [33] and Lewis et al. [34] chose their fentanyl conversion factors based on review of neonatal pain management literature. However, Lewis et al. did not cite a source for their methadone conversion factor. Rana et al. [40] referenced a study of pediatric patients when citing their methadone conversion factor but did not cite a reference for their fentanyl conversion factor.

### Types of opioids requiring conversion

Studies chose to convert between opioids that were relevant to their research goals. All studies converted to morphine milligram equivalents (MMEs), except for one which converted to methadone milligram equivalents to report their findings [41]. Of the 13 studies converting to MMEs, seven converted to intravenous MMEs [32–35, 39, 40, 42], and six converted to oral MMEs [36–38, 43–45].

After morphine, the most common opioid to be included in conversions was fentanyl, present in 11 studies [32–35, 37–40, 42–44]. Six studies included conversions to PO methadone [34, 36, 40, 41, 43, 45], five studies included conversions to PO oxycodone [32, 37, 39, 43, 44], and four included IV hydromorphone conversions [34, 35, 37, 43]. Two studies included hydrocodone [37, 43]. Diluted tinctures of opium [34], buprenorphine [36], and IV nalbuphine [39] were each mentioned in one study.

### Fentanyl

**Fig. 2:**
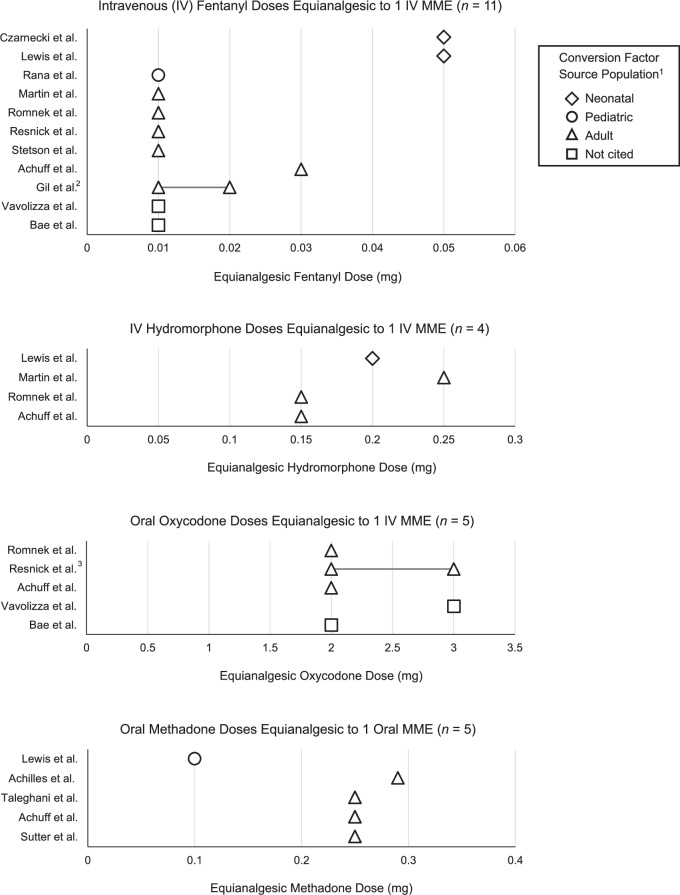
Opioid doses equianalgesic to morphine milligram equivalents (MME). Opioid doses equianalgesic to MME for intravenous fentanyl, hydromorphine, oral oxycodone, and oral methadone. 1 – source populations from which each paper’s cited conversion factors were derived. 2 – Gil et al. [38] did not document equianalgesic dose in text, instead referencing Patanwala [46] who documented fentanyl equianalgesic dose as a range from 0.01 to 0.02mg IV fentanyl equivalent to 1 IV MME. 3 – Resnick et al. [44] did not document equianalgesic dose in text, instead referencing Gammaitoni et al. [47] who documented oxycodone equianalgesic dose as a range from 2 to 3mg oral oxycodone equivalent to 1 IV MME.

Eleven studies converted between fentanyl and morphine [32–35, 37–40, 42–44] with equianalgesic doses shown in Fig. 2. Four studies which referenced adult populations [35, 37, 42, 44] as well as all three studies which did not cite references for fentanyl conversion [32, 39, 40] chose an equianalgesic dose of 0.01 mg IV fentanyl equivalent to 1 mg IV morphine. Achuff et al. [43] cited a larger equianalgesic dose of 0.03 mg IV fentanyl equivalent to 1 mg IV morphine. Gil et al. [38] did not directly document their conversion factor in text and instead referenced Patanwala et al. [46] who cited a conversion factor range of 0.01-0.02 mg IV fentanyl equivalent to 1 IV MME.

Both studies that reported tailoring their choice of equianalgesic dose to the neonatal population [33, 34] chose the largest equianalgesic dose seen in our review: 0.05 mg IV fentanyl equal to 1 mg IV morphine.

### Hydromorphone

Four studies [34, 35, 37, 43] documented equianalgesic conversions between morphine and IV hydromorphone with doses ranging from 0.15-0.25 mg IV hydromorphone equivalent to 1 mg IV morphine (Fig. 2). Three referenced adult populations in choosing their conversion factors [35, 37, 43] and one did not cite any source [34].

### Oxycodone

Of five studies that converted between PO oxycodone and morphine [32, 37, 39, 43, 44] all referenced adult populations when choosing their conversion factors (Fig. 2) with the exception of Vavolizza et al. [32] who did not cite any source. Three studies used 2 mg PO oxycodone equivalent to 1 mg IV morphine [37, 39, 43], and Vavolizza et al. [32] chose 3 mg PO oxycodone equivalent to 1 mg IV MME. Resnick et al. [44] referenced Gammaitoni et al. [47], providing a range of 2-3 mg PO oxycodone equivalent to 1 mg IV MME.

### Methadone

Six studies converted between methadone and morphine [34, 36, 40, 41, 43, 45] (Fig. 2). Three of these used methadone as their only non-morphine opioid [36, 41, 45]. Each of these studies exclusively included patients with exposure to opioids in utero and were focused on medication treatment of neonatal abstinence syndrome (NAS) or neonatal opioid withdrawal syndrome (NOWS). Since the majority of studies which included a methadone-morphine conversion chose to convert to oral rather than intravenous morphine equivalents, Table 2 and Fig. 2 demonstrate the equianalgesic doses of methadone equivalent to 1 mg PO morphine. Rana et al. [40] were excluded from Fig. 2 due to their study not including any conversion between IV and PO morphine equivalents, therefore reviewers were unable to assume any conversion factor between PO methadone and PO morphine from this study.

Of all studies involving methadone conversion, four referenced adult populations [36, 41, 43, 45], one referenced a pediatric population [40], and one referenced a neonatal population [34]. All studies which referenced adults clustered around 0.25 mg PO methadone equivalent to 1 mg PO morphine, an equianalgesic dose consistent with the CDC’s Opioid Prescribing Guideline and MME conversion table [10]. Rana et al. [40] chose to use 1 mg PO methadone equivalent to 1 mg IV morphine. The remaining study, Lewis et al. [34], referenced a neonatal population in their choice of fentanyl conversion factor, but did not cite a reference for their methadone conversion. In fact, they chose the smallest equianalgesic dose of our review for their conversion: 0.1 mg PO methadone to 1 mg PO morphine.

## Discussion

This systematic review highlights the lack of consensus and underscores the need for standardization in reporting of opioid exposure in neonates and infants. Of 30 studies of patients <1 year of age which endorsed use of opioid equivalents in their methodology, only sixteen met initial inclusion criteria in our study by documenting in text or citing sources to explain the calculations performed to reach reported opioid equivalents. Considering the notable variation in conversion factors chosen between the studies included in our analysis, it is unclear how the excluded studies carried out their methodologies.

Inaccurate conversion between opioids may lead to either inadequate pain management or overmedication, with significant medical consequences in any patient population. These consequences are even more pronounced in neonates and infants who have distinct body physiologies and are especially prone to both short term adverse outcomes such as hypotension [22] and respiratory depression [21, 22]. In addition, studies demonstrate negative long-term outcomes such as abnormal brain development [48], neurodevelopment [7–9] and socialization [8] related to non-optimized opioid exposure.

Considering the two studies that documented consideration of neonatal physiology when choosing conversion factors [33, 34], each consistently chose equianalgesic doses that would result in smaller equivalent doses of the longer-acting opioid relative to the studies which referenced adult populations. Czarnecki et al. [33] called their choice of conversion factor “conservative” and emphasized the impact that conversion factors have on results, but ultimately reaffirmed that true equianalgesic conversion between fentanyl and morphine is unknown in neonates.

None of the records included in our analysis were designed to study the conversion between opioids–each referenced a conversion factor only to quantify overall morphine exposure in their study population. However, we can look to these studies’ citations to better understand the work that has been done to elucidate opioid conversion factors in infants <1 year of age.

In preparation of this report, one study of neonates was found which attempted to prove a conversion factor between two opioids by direct comparison of opioid exposures. In 2012, Jeffries et al. [28] retrospectively studied the IV morphine or PO methadone used to initiate opioid wean after prolonged IV morphine exposure (5-10 days) in the NICU. They then calculated a conversion ratio based on these initial morphine or methadone doses prescribed, yet ultimately concluded that further research was needed to determine an optimal conversion factor between morphine and methadone. Further, this study was limited by studying a retrospective, not prospective, set of data.

All other studies that referenced neonatal or infant populations and explained their choice of conversion factor only inferred equianalgesic doses based on studies of the independent, individual pharmacokinetic and pharmacodynamic profiles of the opioids being converted [49–52]. Without direct comparison, true conversion between opioid types cannot be evidentiarily based.

Most studies which referenced adult conversion factors, and the majority of studies overall, reported the same equianalgesic dose of 0.01 mg IV fentanyl equivalent to 1 mg IV morphine [32, 35, 37–41, 44] and the same equianalgesic dose of 2 mg PO oxycodone equivalent to 1 mg IV morphine [37, 39, 43, 44]. With respect to fentanyl and oxycodone, most published literature uses a conversion factor standardized to adults. No clear trends exist in equianalgesic opioid doses of IV hydromorphone or PO methadone, though these comparisons were limited by even smaller sample sizes.

Current published literature on infant opioid administration has established a precedent of using adult literature to perform opioid conversions. While this leads to consistency and reproducibility in research, it may limit our ability to translate findings into clinical practice and safely implement opioid stewardship in infant care. Our findings strongly support the need for pharmacokinetic studies of opioids within the pediatric population and specifically the neonatal and infant populations. Further investigation into how prematurity and low birthweight affect pharmacokinetics are needed as these infants may be especially vulnerable to negative outcomes of opioid exposure and may therefore require different conversion factors [7, 8, 17]. Additionally, many infants such as those on ECMO or term infants requiring surgery are exposed to opioids for prolonged periods. Using an incorrect or unvalidated opioid conversion factor could have a substantial impact on in-hospital outcomes and long-term neurodevelopment [7, 48, 53, 54]. Ultimately, pediatric professional societies and funding agencies dictating quality of care and funding priorities must encourage use of pediatric-specific metrics for opioid dosing as quality checks for novel research. As the role of technology grows in healthcare and interest increases to employ artificial intelligence in our field, more infant-specific opioid dosing literature is needed to ensure that the tools embedded in our electronic health records are safe and accurate.

### Limitations

Our study’s primary limitation is its combined inclusion of premature, neonatal, and infant populations when selecting studies for analysis. As described in the background, physiology throughout the first year of life is dynamic. Neonates comprise a distinct group with physiology significantly different from infants older than 1 month and premature infants face a host of additional physiological considerations different from the typical neonate born at term.

## Conclusions

Equianalgesic ratio methodology used to calculate MMEs is often not documented nor cited in published literature. Most neonatal literature uses the same equianalgesic ratios that are widely accepted in adults. When neonatal physiology was considered, the equianalgesic doses chosen favored smaller equivalent doses of longer-acting opioids. Future pharmacokinetic studies in the neonatal population are needed to develop evidence-based consensus required to expand opioid stewardship to hospitalized infants.
